# Functional Divergence of the *Tribolium castaneum engrailed* and *invected* Paralogs

**DOI:** 10.3390/insects14080691

**Published:** 2023-08-04

**Authors:** Summer Blunk, Hector Garcia-Verdugo, Sierra O’Sullivan, James Camp, Michael Haines, Tara Coalter, Terri A. Williams, Lisa M. Nagy

**Affiliations:** 1 Molecular and Cellular Biology, University of Arizona, Tucson, AZ 85721, USAhdg1@arizona.edu (H.G.-V.); sierra.osullivan@dmu.edu (S.O.);; 2 Biology Department, Trinity College, Hartford, CT 06106, USAterri.williams@trincoll.edu (T.A.W.)

**Keywords:** *engrailed*, invected, segment-polarity, sequential segmentation, *Tribolium*, gene paralogs

## Abstract

**Simple Summary:**

The *engrailed* (*en*) and *invected (inv)* paralogs play a fundamental role in arthropod segmentation. Previous research suggests that knockdown of either *en* or *inv* in sequentially segmenting insects leads to an unexpected and variable loss of segments but does not mimic the segment polarity defects seen in *Drosophila en* mutants; the consequences for segmentation when both paralogs are lost have not been reported outside of *Drosophila*. We analyzed the phenotypes of single and double knockdowns in the flour beetle *Tribolium castaneum*. Unlike *Drosophila*, *inv* knockdowns are inviable, consistent with a functional divergence of the paralogs between *Tribolium* and *Drosophila*. We find the *Tribolium* paralogs are redundant and act synergistically to pattern trunk appendages and segments. The most common *Tribolium* double knockdown results in small, limbless larvae that suffer a loss of a portion of each trunk segment that shares characteristics with segment polarity mutants in *Drosophila*. Some of the double knockdown embryos arrest development before germband retraction, consistent with an underexplored early function for *en* and *inv* in the regulation of cell proliferation or death in sequentially segmenting insects.

**Abstract:**

*Engrailed (en)* and *invected (inv)* encode paralogous transcription factors found as a closely linked tandem duplication within holometabolous insects. *Drosophila en* mutants segment normally, then fail to maintain their segments. Loss of *Drosophila inv* is viable, while loss of both genes results in asegmental larvae. Surprisingly, the knockdown of *Oncopeltus inv* can result in the loss or fusion of the entire abdomen and *en* knockdowns in *Tribolium* show variable degrees of segmental loss. The consequence of losing or knocking down both paralogs on embryogenesis has not been studied beyond *Drosophila*. To further investigate the relative functions of each paralog and the mechanism behind the segmental loss, *Tribolium* double and single knockdowns of *en* and *inv* were analyzed. The most common cuticular phenotype of the double knockdowns was small, limbless, and open dorsally, with all but a single, segmentally iterated row of bristles. Less severe knockdowns had fused segments and reduced appendages. The *Tribolium* paralogs appear to act synergistically: the knockdown of either *Tribolium* gene alone was typically less severe, with all limbs present, whereas the most extreme single knockdowns mimic the most severe double knockdown phenotype. Morphological abnormalities unique to either single gene knockdown were not found. *inv* expression was not affected in the *Tribolium en* knockdowns, but *hh* expression was unexpectedly increased midway through development. Thus, while the segmental expression of *en/inv* is broadly conserved within insects, the functions of *en* and *inv* are evolving independently in different lineages.

## 1. Introduction

It has long been known that most insect species add their segments sequentially, in contrast to the near-simultaneous process in dipteran insects such as *Drosophila*. The discovery of the developmental genetic network that regulates *Drosophila melanogaster* segmentation has opened the door to the comparative exploration of the molecular basis of segmentation in insect species lacking the same genetic tools. The *Drosophila* segmentation genes have become key tools for understanding the evolution of differences in the mechanisms of segmentation. Most, but not all, of the *Drosophila* segmentation genes, are readily identified across species, yet many of their expression patterns, functions, and regulatory interactions vary throughout arthropods (reviewed in [1,2]). One exception to this pattern is the class of genes referred to as segment polarity genes. Particularly noteworthy is the remarkable conservation of the expression patterns of the segment polarity genes *engrailed (en)* and/or *invected (inv)* that encode paralogous homeodomain-containing transcription factors.

In *Drosophila*, *en* and *inv* function to establish and maintain the posterior compartment in segments and appendages throughout development [3,4,5]. *en* and *inv* are also known to function in neurogenesis [6,7], axon targeting [8] wing venation, and butterfly wing coloration patterning [9,10,11,12]. Their expression has been widely examined, most frequently using the 4D9 antibody that typically recognizes the homeodomain of both proteins [13]. In all species examined, 4D9 expression is detected in a narrow stripe in the posterior compartment of each segment [14,15,16,17,18,19,20,21,22]. In those species for which both *en* and *inv* expression has been examined separately, their expression co-localizes during segmentation, with only minor variance in the onset of expression in antennal and mandibular segments in one species [15,16,23,24]. The timing of their embryonic expression is thought to coincide with the ‘phylotypic stage’ of insects—the segmented germband [1].

Further support for the conservation of their function came from comparative analyses of an early acting feedback loop between *wingless (wg)*, *en,* and *hedgehog (hh)* known to maintain the architecture of the *Drosophila* segment. *wg* expressing cells are restricted to the anterior segmental compartment and secrete *wg* protein toward the posterior which maintains *en* in the cells within the posterior compartment. In turn, *en* regulates *hh* to maintain *wg* in the anterior compartment. Failure to maintain this feedback loop results in a failure to maintain the segment boundaries. Later, *wg* and *en* function independently [5,25,26,27,28,29]. This feedback loop is expected to be conserved in other species, and its conservation is supported by the fact that transplantation of *en*-expressing cells creates new boundaries when juxtaposed to cells in the anterior of *Oncopeltus* segments [30] and more directly by ectopic expression of *wg* via a baculovirus vector in *Tribolium* embryos that leads to induction of *en* expression in adjacent cells [31]. In addition, embryos and larvae with the most severe reduction in the function of *Tribolium hh* and *wg* share similarities with one another [31].

Given the widespread conservation of patterns and timing of expression during a conserved embryonic stage, as well as support for conserved regulatory interactions, it was generally assumed that the function of *en/inv* genes would also be conserved. Consequently, their function in early segmentation, either in single or double loss of function or knockdown experiments, has not been widely explored. Beyond *Drosophila*, there is a single report of *Tribolium en* knockdowns [32] and a single report of *Oncopeltus fasciatus inv* knockdowns [14], (see Supplemental Data Note). In both cases, the results differ from that expected from *Drosophila*. *Drosophila en* mutants are embryonic lethal and have an unusual phenotype displaying features that affect the cuticular pattern in both a pair-rule and segmental manner [4,33]. By contrast, *Tribolium castaneum en* knockdowns result in cuticles with incomplete or irregular segment boundaries, missing anywhere between 1–11 trunk segments [32]. Null mutations in the other *Drosophila en* family paralog *inv* are viable [34,35]. However, when *inv* is knocked- down in *Oncopeltus fasciatus*, the phenotype varies from segment fusions and poorly demarcated boundaries to larvae in which the entire abdomen is lost or fused, suggesting segment development from the growth zone might have been affected [14]. The *Oncopletus inv* and *Tribolium en* knockdown data suggest that *en* and *inv* function in segmental patterning may vary from their described roles in *Drosophila*.

The *en* gene has long served as a model for the fate of gene duplications [36]. Duplications within metazoan lineages have resulted in gene families of one to four copies, in which the paralogs have different degrees of divergence in expression and function [12,23,37,38]. For example, in zebrafish, subfunctionalization of *en* duplicates led to differential expression in pectoral appendages and neurons [36]. In contrast to this widespread divergence of function between *en* family paralogs, the degree to which the hexapod *en/inv* gene duplication serves redundant functions in segmentation is unknown. When both *en* and *inv* functions are lost in *Drosophila*, segmentation defects are more severe than those that result from the loss of either gene alone. The double mutant results in truncated embryos and a ‘lawn of denticles’, with no apparent segmentation, indicating redundancy of *en* and *inv* in maintaining the structure of the segment in the fruit fly [34]. The effect of the loss or reduction of function of both genes on segmentation has not been tested in any insect other than *Drosophila.*

In *Tribolium*, the consequences of a reduction in *inv* function have yet to be reported, and the consequences for embryonic development due to the knockdown of both *en* and *inv* have not been explored. Through single and double knockdowns of *en* and *inv* with embryonic RNAi (eRNAi), we find support for the previously reported *en* knockdown phenotypes and propose a novel interpretation for the loss of segment phenotype. Our double knockdowns have disruptions of both anterior-posterior and dorsal-ventral boundaries, segment fusions, as well as a complete loss of distal leg elements. Severely affected embryos that develop cuticles are dramatically reduced in size; these highly contracted embryos retain their full segment number within the CNS, as confirmed with antibody staining. Our results suggest that *en* and *inv* serve redundant functions in gnathic and leg development, and the late maintenance of segment integrity. Interestingly, a significant portion of the dsRNAi-treated embryos fail to form cuticles at all, with phenotypes consistent with a failure to grow. The *Tribolium inv* or *en* single RNAi knockdowns result in phenotypically similar larval defects that are, for the most part, significantly less severe than the RNAi knockdown of both *en* and *inv*. We did not find evidence for functions unique to either paralog. Our results are consistent with a model in which the *en/inv* paralogs act synergistically to redundantly pattern the embryo.

## 2. Materials and Methods

### 2.1. Husbandry, Egg Collections, and Injection Preparations

*Tribolium castaneum* beetles (Strain GA-1) were maintained on whole-wheat flour supplemented with yeast (5% whole volume) at 30 °C, 40–60% humidity. To collect embryos for injection, adult beetles were incubated at 30 °C on white flour for one hour. Eggs were collected using stainless-steel mesh sieves (710 μm and 300 μm). Embryos were prepared for injections after incubating for 4 h at 30 °C. They were transferred to a handmade dechorionation apparatus constructed from a 50 mL plastic centrifuge tube with a large hole cut in its lid and a piece of mesh tightly secured to allow the eggs to be agitated in a 5% bleach solution for 2 min and then rinsed in distilled water 3 to 5 times. Eggs were transferred to a microscope slide using a 5/0 paintbrush and oriented to enable the injection needle to enter the lateral flank of the egg. The dechorionated eggs adhere sufficiently to the slide to allow resistance to the injection needle without further manipulation.

### 2.2. Cloning and dsRNA Synthesis

**Figure 1:**
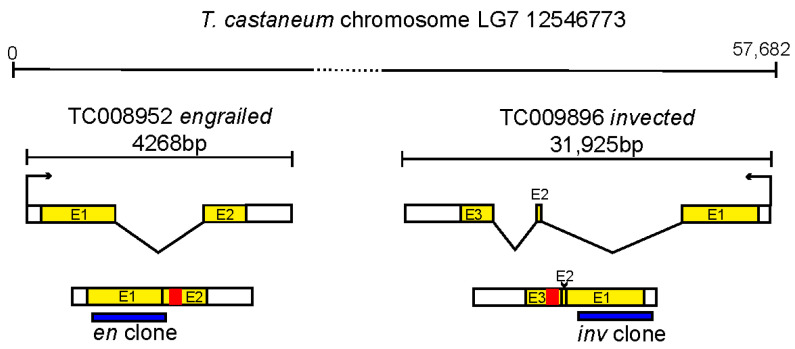
Description of clones used in this study. Location of dsRNA fragments within the *en* and *inv* genes. The *Tribolium en* and *inv* gene are present in a closely linked tandem duplication shown at the top (as previously described in [24]). Exons (E1,2,3) are colored yellow, the homeobox encoding region is colored red, location of the regions cloned and used for dsRNA experiments is shown in blue.

PCR primers amplified a 477 bp region of *en* Tc-008952 and a 627 bp of *inv* Tc-009896 (see Supplemental Note on identification of *en/inv* orthologs from genome databases). Amplified fragments were designed to exonic regions, avoiding the homeobox (Figure 1). To avoid off-target sequences for *inv* cloning, we performed a BlastN search on NCBI of the cDNA for our genes, choosing the algorithm “somewhat similar sequences”. We examined the hits for stretches of base pairs longer than about 15 bp and chose our clones to exempt potential overlap with known genes, e.g., in the highly similar homeodomain area. Our potential *Tc-inv* RNA fragments were also run through the Deqor software, and no off-target hits were identified [39]. PCR primers were designed with an optimal size of roughly 500 bp (as defined by [40]). For the *Tc-en* RNA fragment, we chose to use a nearly identical fragment used by [32].

The desired gene fragments were amplified via PCR from genomic DNA. Amplified fragments were cloned into the pSC-A-amp/kan PCR cloning vector (Agilent Stratagene TA Cloning Kit, Santa Clara, CA, USA) and verified by sequencing (UA Genomics Core, Tucson, AZ, USA). Standard PCR reactions were conducted using OneTaq reagents from New England Biolabs and primers designed to create a T7 primer sequence on either end of the *Tc-en* and *Tc-in* clones (for downstream dsRNA synthesis). dsRNA was synthesized and purified with the T7 MEGAscript kit (Ambion, Carlsbad, CA, USA). dsRNA solutions used for injection included 0.5, 1.0, 1.5 and 2.0 µg/µL each of *Tc-en* and *Tc-in*.

### 2.3. Microinjections and Needle Preparation

A Narishige Micromanipulator was used in conjunction with a 1.0 mm × 100 mm thin wall glass capillary needle (TW100F-4; WPI, Inc., Sarasota, FL, USA) shaped using a Model P-97 Sutter Instruments Co., (Novato, CA, USA) needle puller. Needles were broken under a dissecting microscope with fine-tip forceps. The injections were carried out using an aspirator tube assembly (Sigma, St. Louis, MI, USA) fitted with a solution-filled 3.5′ glass capillary tube (Drummond, Birmingham, AL, USA). The nitrogen gas pressure balance and pressure are first set to pBal = 0.5 PSI and pOut = 28 PSI and adjusted as needed. Microinjection was performed under a Leica dissecting scope. For embryonic injections, embryos were collected for one hour and then aged at 30 °C for 3 h prior to processing for injection. The embryos were injected without any covering. All solutions were injected into the lateral flank of each egg. Once injections were complete, embryos were kept on the injection slide, stored on petri dishes filled with 1% agarose inside a closed large Tupperware container, and raised to the desired age at 30 °C. To raise the embryos to hatchlings the Tupperware containers were opened after the 3rd day of incubation to decrease the humidity in the container.

### 2.4. qPCR Analysis

For *Tc-en* qPCR experiments, embryos were injected with either 1 or 2 ug/uL *Tc-en* at 4 h AEL and collected at later developmental stages, roughly (24, 31, 48, 50 h AEL). Total RNA was isolated from 50 or more buffer-injected controls and dsRNA-injected eggs using Trizol (Invitrogen), according to the manufacturer’s instructions. The aqueous phase was purified using the NEB Monarch RNA mini-prep kit (NEB #T2010). 10 ng of RNA was used in each reaction of the NEB Luna Universal One-Step RT-qPCR kit (cat # E3005). RT-qPCR reactions were carried out in triplicate, and melting curves were examined to ensure single products. Results were quantified using the CFX Maestro software from Biorad using the ‘‘delta-delta Ct” method and normalized to *Histone 3 (H3)* transcript levels (Supplemental Figure S2). Primer sequences used are: *H3* F: 5′-CTGCCCTTCCAGAGATTGGT-3′; *H3* R: 5′-GAACAGACCCACGAGGTACG-3′; *en* F: 5′-CGCAGGGACTCTACAACCAC-3′; en R: 5′-CGAGATTTGCCTTCGCTCTC-3′; *inv* F: 5′-GCAAGCCGAAGAAGGTTGTG-3′; *inv* R: 5′-TTCTTGACTCGCCTGGTTCG-3′; *hh* F: 5′-CACTGAAGGACGCATCGGAA-3′; hh R: 5′-GGTTCATCACCGAAATCGCC-3′.

### 2.5. Cuticle Preparation

Samples were mounted in 1:1 of Sigma Aldrich Lactic Acid ACS reagent ≥85% and Hoyer’s mounting solution and placed on a heat block set to 60 °C for 12–24 h. Samples were imaged using a Zeiss Axioplan2 and captured with AxioVision 4.8 software at either 10 or 20×. DIC/Nomarski imaging was performed using high-contrast settings.

### 2.6. Statistical Analysis

The statistical significance of the mean cuticle length was tested by one-way ANOVA, and statistical significance was defined as *p* < 0.05. The beetle categories were based on knockdown type and concentration of knockdown. Two groups were compared at a time to determine if there was a difference in mean length.

### 2.7. Embryo Fixation and Immunohistochemistry

Embryos were dechorionated in 5% bleach for 2 min, washed with distilled water to fully dilute the remaining bleach, and fixed in a 1:1 (*v*/*v*) ratio of n-heptane and 4% formaldehyde, for ~45 min. The fix solution was removed, replaced with ice-cold 100% MeOH, and shaken vigorously by hand for 2 min. to encourage devitellinization. Embryos that fell to the bottom were separated as devitellinized; embryos remaining at the interphase were either sonicated, dissected, or left as whole embryos and stored in 100% methanol. Immunohistochemistry followed the protocol as described in [13]. Antibodies used were: 4D9—Engrailed/Invected (DSHB) [13]; FP6.87-Ubx-abdA DSHB) [41]. We assume 4D9 detects both En and Inv proteins and FP6.87 detects both Ubx-abdA, although that has not been directly tested in *Tribolium*.

## 3. Results

### 3.1. Knockdown of Both en and inv Produces a Range of Morphological Defects

**Figure 2:**
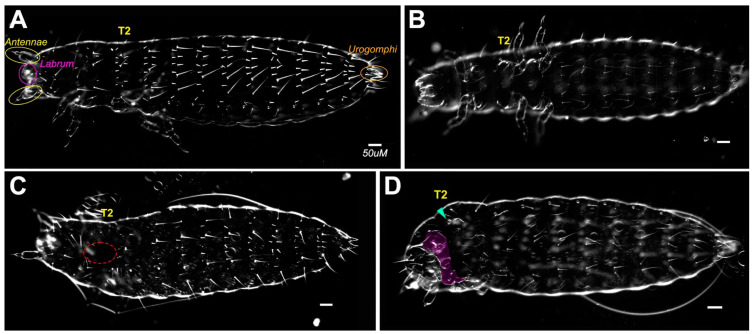
Mild phenotypes resulting from double dsRNA knockdown of *en* and *inv*. (**A**) Dorsal side of buffer injected control; (**B**) ventral side of buffer injected control; (**C**) Category B larva, primarily normal, with incomplete dorsal closure on first and second thoracic segments (outlined in red); (**D**) Category B larva, primarily normal with missing (arrowhead) or abnormal appendages (highlighted in purple). All scale bars = 50 µm.

**Figure 3:**
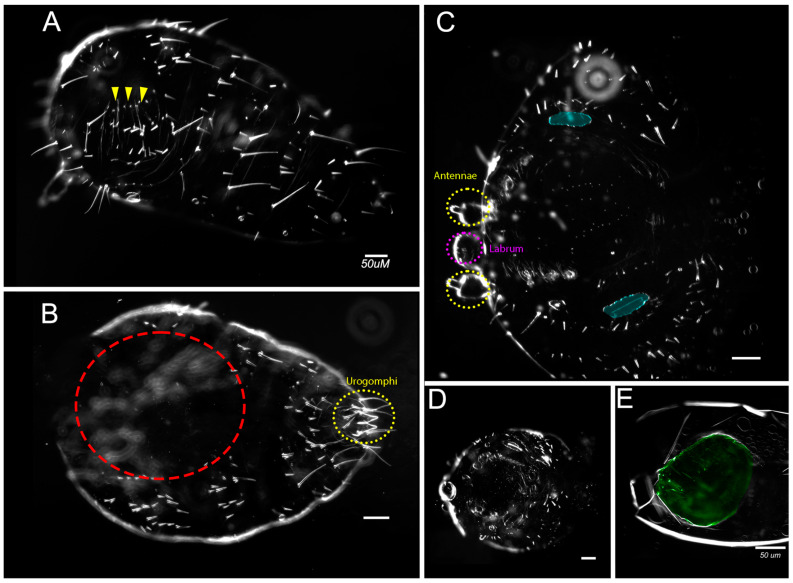
Variety of severe phenotypes resulting from knockdown of both *en* and *inv*. (**A**) Category C larva with fusing and minimized gnathic and thoracic segments (arrowheads); (**B**) Category C larva with a reduced number of segments and incomplete dorsal closure (red circled area) but complete urogomphi (circled in red). (**C**) Category D asegmental larva with fused spiracles (blue). Moreover, note the absence of long bristles, mouthparts, and legs; antennae (circled in yellow) and labrum (circled in purple) were present. (**D**) Category D asegmental larva with similar cuticular features to C but more complete dorsal closure; (**E**) Category E larvae with secreted cuticle and minimal cuticular features. All larvae are oriented anterior to the left. Larvae had been injected with 0.5 µg/µL of both *Tc-en* and *Tc-inv*, except for C, which was injected with (1 µg/uL each of *Tc-en* and *Tc-inv*). Scale bar = 50 µm.

**Figure 4:**
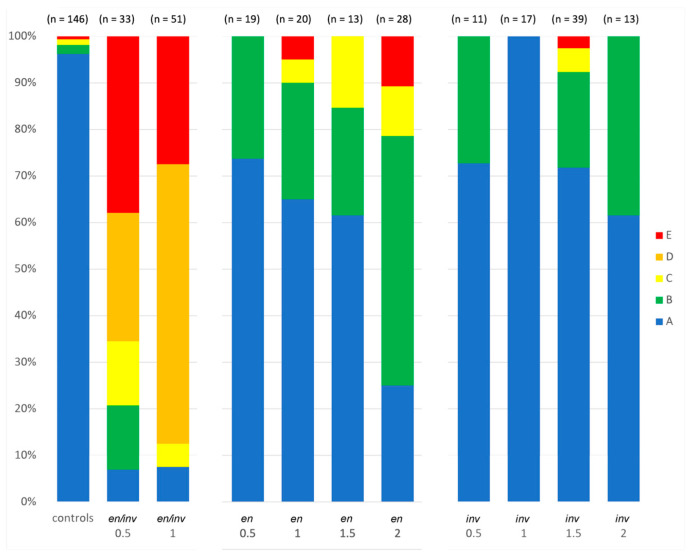
Phenotypic categories resulting from increasing concentration of *Tc-en/inv* dsRNA injected. Category A: phenotypically normal; Category B: fully segmented, primarily normal with incomplete dorsal closure on the anterior thorax, reduced limbs, and minor disruptions of bristle pattern; Category C: larvae with fused and/or missing segments, a greater degree of incomplete dorsal closure, and missing appendages; Category D: asegmental larvae with fused tracheal pits, reduced bristle pattern, and failed dorsal closure; Category E: asegmental, unhatched larvae with minimal or no cuticular features. Sample sizes are indicated at the top of each bar; concentrations in µg/µL of dsRNA injected of either *en/inv*, *en,* or *inv* dsRNA are indicated below the bars.

The larval cuticles resulting from *Tc-en/inv* dsRNA-injected embryos showed substantial phenotypic variability. To represent that variability, we grouped larvae into categories reflecting the severity of segment and appendage loss, bristle pattern, segment fusion, and dorsal closure defects (Figure 2, Figure 3 and Figure 4).

#### 3.1.1. Segment Number

Larvae varied from asegmental to fully segmented, although greater than 75% of the double knockdowns were asegmental (Figure 4). Category A larvae were phenotypically normal (Figure 2A,B). Category B larvae were fully segmented (Figure 2C,D). Category C larvae (Figure 3A,B) were missing between 1–9 trunk segments, with posterior segments less affected. Categories D and E (Figure 3C–E) were asegmental and included unhatched larvae.

**Figure 5:**
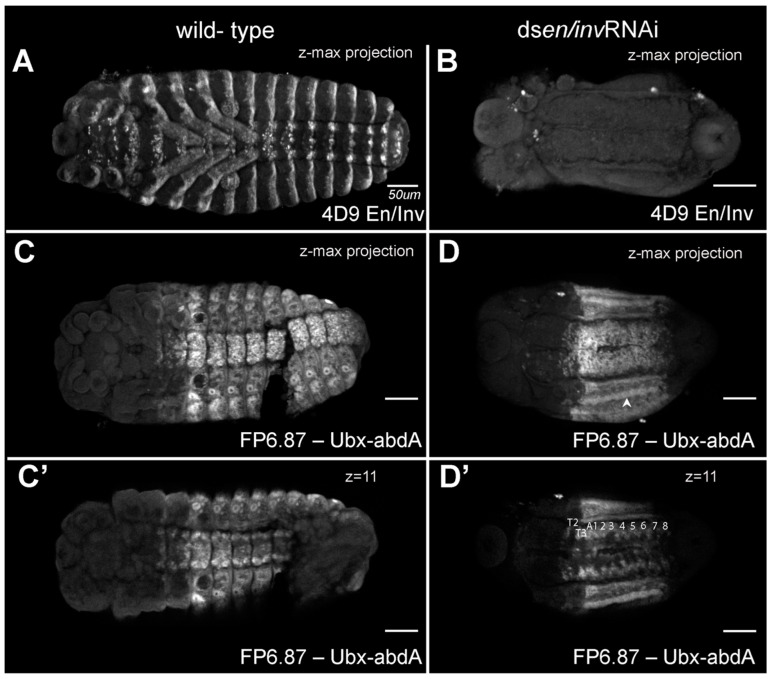
En/Inv and Ubx-adbA protein expression in wild type and *en/inv* RNAi knockdowns. (**A**) En/Inv expression in wild-type and (**B**) *en/inv* RNAi knockdowns. (**C**,**C’**) Ubx-abdA expression in wild-type embryos. (**D**,**D’**) Ubx-abdA expression in *en/inv* RNAi knockdowns. Ubx-adbA protein expression extends from the posterior of the third thoracic segment through 8 abdominal segments in both wild-type and knockdown embryos. (**C’**,**D’**) The expression is shown in a single focal plane. The arrowhead in (**D**) points to fused spiracles. Both WT and knockdown embryos were reared to 48–50 h AEL. Scale bars = 50µm.

To examine segmentation and to what degree *en/inv* were knocked down in the extremely reduced phenotypes, we fixed embryos at stages prior to the secretion of cuticle and examined the expression of En/Inv proteins using the 4D9 antibody ([13]; Figure 5A,B) and expression of Ultrabithorax/abdominal-A using the FP6.87 antibody [41]; Figure 5C–D’). Wildtype embryos (48–50 h AEL) show standard En/Inv stripes in the posterior of each segment; expression can be seen in the (3) gnathal, (3) thoracic, and (10) abdominal segments, as well as in all appendages. The double knockdown is much smaller and has no detectable En/Inv stripes. The CNS tissue occupies a more extensive amount of the embryo width, and the tissue has not begun to close dorsally (not shown in image). In the Ubx/abd-A specimens, a ridge of cells strongly expressing the Ubx/abd-A antigen (arrowhead in B) runs down the lateral flank of both sides of the embryo, likely correlating to the spiracles (bright expression surrounding each tracheal pit in the control). A similar ridge appears in every embryo analyzed and seems to delineate the ventral midline tissues (which have visible segmental structures) from the lateral flanks (with smoothened appearance), suggesting that segmental expression is still detectable in the CNS but segments in the lateral flank have fused/lost segmental boundaries. We compared individual z-slices to the z-max projection, confirming that at least 8 abdominal segments are present in these animals; the non-expressing tissue posterior to the final segment was identified as the protruding hindgut, slightly curled ventrally.

#### 3.1.2. Larval Length

**Figure 6:**
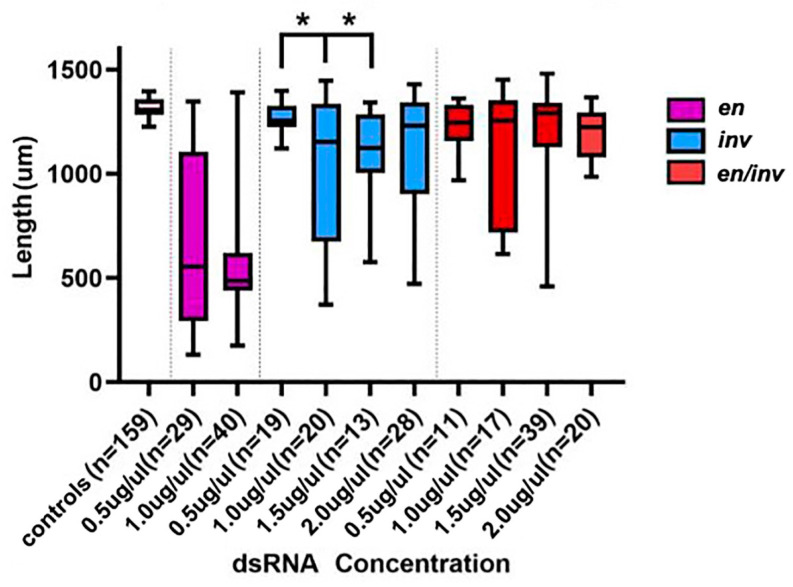
Reduction in length as a function of dsRNA injected. All concentrations of dsRNA used in single or double knockdowns led to a significant reduction in length (*p* < 0.05) relative to controls. Within each group, only *en* 0.5 µg/µL relative to either *en* 1.0 µg/µL or *en* 1.5 µg/µL led to significant differences in length, indicated with an asterisk. No *inv* or *en/inv* dsRNA concentrations led to larvae significantly different in length from one another. dsRNA concentrations are shown in µg/µL.

**Figure 7:**
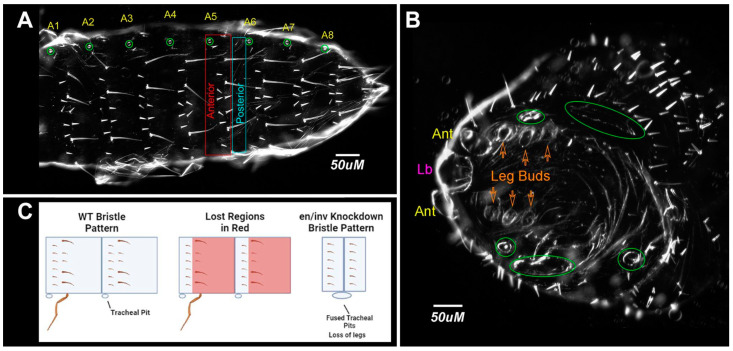
Model for loss of repeated segmental elements and segment fusions. (**A**) Dorsal view, buffer-injected control. Spiracles are circled in green; (**B**) *en/inv* dsRNA injected (1 µg/µL) category D larvae with repeating rows of short bristles missing intervening regions of the naked cuticle. Fused spiracles are circled in green. Ant, antennae, Lb, labrum. (**C**) Diagram of loss of cuticular phenotypes in the severely affected double knockdowns.

Larvae resulting from the *Tc-en/inv* dsRNA injections were significantly shorter (using *p* < 0.05) than buffer-injected controls along the AP axis (Figure 6). The reduction in size along the AP axis can be attributed to several independent features of the phenotype: (1) Intrasegmental tissue loss: the loss of bristle and naked cuticle pattern elements suggests a loss of a significant portion of many, if not most, of the segments (Figure 7). (2) Segment fusions: fusions were detected by the presence of fused tracheal pits on the ventral and lateral surfaces (Figure 3C,D, Figure 5B,D and Figure 7B). Further evidence of segment fusion was seen dorsally, where rows of bristles from two to four segments were observed to converge along the midline (not shown). Posterior segments fused less frequently: in category C, the segment fusions left the two most posterior abdominal segments unaffected or much less affected than the anterior abdominal and thoracic segments (Figure 3A,B). (3) Reduction in the size of the head: The head was severely reduced in size in all but Category B embryos.

#### 3.1.3. Appendage Formation

Gnathal and thoracic appendages were either lost or significantly reduced in size in all categories (Figure 3, Figure 5 and Figure 7). The labrum and antennae were present but reduced in size (Figure 3, Figure 5 and Figure 7). Urogomphi were unaffected in all but the most severely affected larvae (e.g., Figure 3C). Appendage loss was independent of segment loss-appendages were lost, or present only as small buds, even in embryos with normal segmentation.

#### 3.1.4. Bristle Patterns

Bristle patterns highlighted abnormalities in segment formation. A hallmark feature of Category D phenotypes was the repeated basiconic sensilla (as described in [41]) covering the lateral and dorsal cuticle, atypically uninterrupted by the naked cuticle (Figure 3C and Figure 7). In Category C larvae, rows of bristles were atypically discontinuous along the dorsal midline (Figure 3B). Category E (Figure 3E) larvae were covered in primarily naked cuticles and only a few bristles of uncertain segmental origin could be identified.

#### 3.1.5. Dorsal Closure

The majority of larvae resulting from the *Tc-en/inv* eRNAi also failed to complete dorsal closure. The least affected larvae failed to close along the dorsal midline of the first thoracic segment (Figure 2C). The most severely affected larvae failed to close at any position along the AP axis. This could also be seen in the examination of late-stage embryos.

#### 3.1.6. Failure to Form Cuticle

**Figure 8:**
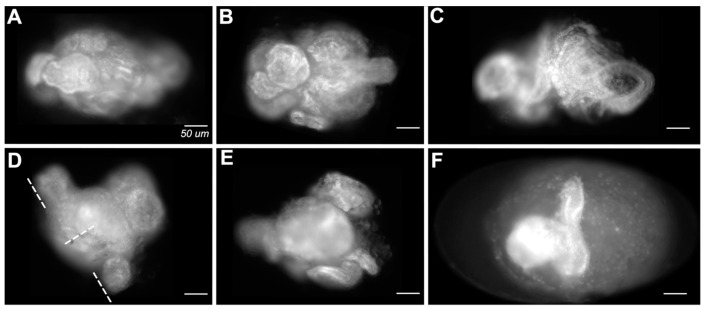
Representative images of embryos that failed to secrete cuticle. (**A**–**F**) Embryos stained with DAPI. Embryo (**F**) is shown within the vitelline membrane, the remaining panels are cropped to isolate the embryo. Dotted lines in (**D**) indicate unusual orthogonal axes, also shown visibly in (**F**). Scale bars = 50 µm.

The most extremely affected embryos (Figure 8; on average 22.5% of double knockdowns; 10.25% of single knockdowns) lacked cuticles altogether. Embryonic tissue could be identified in these embryos but was frequently curled and spiraled within the vitelline membrane. Some embryos had an obvious anteroposterior polarity but had no distinguishable axial characteristics. Some appeared to have formed extensions orthogonal to the A/P axis (Figure 8D,F).

### 3.2. Single Knockdowns

**Figure 9:**
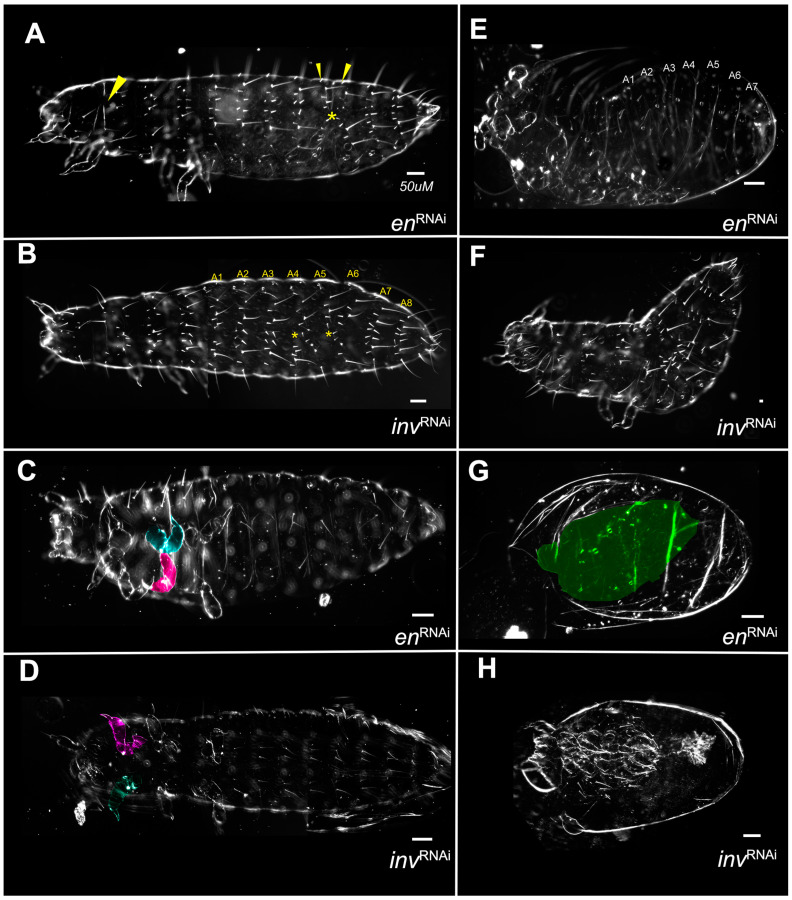
No qualitative differences between *Tc-en* and *Tc-inv* single knockdowns. (**A**) 2.0 µg/µL *Tc-en* dsRNA injected larva with abdominal bristle fusions (yellow* and small arrowheads) and minor failure to complete dorsal closure (large arrowhead); (**B**) 1.5 µg/µL *Tc-inv* dsRNA injected larva with fusing segments A4-A5 and A5-A6 (yellow*); (**C**) 2.0 ug/uL *Tc-en* dsRNA injected larva with misshapen limbs (T2 legs colored); (**D**) 0.5 µg/µL *inv* dsRNA injected larva with misshapen limbs (T1 legs colored); (**E**) 2.0 µg/µL *Tc-en* larva with misshapen limbs, failed dorsal closure and missing abdominal segment; (**F**) 1.5 µg/µL *Tc-inv* dsRNA injected larva with segmental fusion, missing abdominal segment; (**G**) 2.0 µg/µL *Tc-en* dsRNA injected amorphous cuticle highlighted in green; (**H**) 1.5 µg/µL *Tc-inv* dsRNA injected larva very reduced in size, with the vestigial abdomen.

Single knockdowns of either *Tc-en* or *Tc-inv* resulted in similar phenotypes, although the *inv* dsRNA-injected individuals were more frequently normal under all concentrations of dsRNA injected (Figure 4). Even at the highest concentrations of injected dsRNA for either gene, the larvae were primarily normal morphologically, with minor patterns of bristle fusions (Category A,B; Figure 9A,B). More severe phenotypes included primarily irregularly shaped appendages, with a bloated appearance (Figure 9C,D). The more severe phenotypes were overall smaller in size, with greater deformation of the appendages, and missing or fused segments (Figure 9E,F,H). A single *inv* dsRNA injected larva developed nearly normal gnathic and thoracic appendages but lacked an abdomen Figure 9H). Unlike the double knockdowns, except in the most extreme phenotypes (Figure 9G) appendages were present. Category D phenotypes (Figure 3C,D and Figure 7B) were not observed in the single knockdowns.

#### 3.2.1. *Asegmental embryos* at High dsRNA *Tc-en* or *Tc-inv* Concentrations

In double knockdowns, the loss of all segmentation, bristle pattern, and morphology was observed in approximately 28% of samples injected with 1 µg/µL *Tc-en/inv* dsRNA (Category E). This phenotype was not observed in single gene knockdown larvae resulting from 0.5 or 1 µg/µL *Tc-en* or *Tc-inv* dsRNA injections, but became more prevalent with increasing concentrations of dsRNA injected: at 1.5 µg/µL *Tc-inv* dsRNA injected 3% of the sample were category E, at 2.0 µg/µL *Tc-en* dsRNA injected 11% of the sample were Category E. These larvae were inviable and lacked visible morphological structures. However, the cuticle preparations of these samples revealed that the cuticle was present, frequently with an axial polarity and a few random bristles (Figure 9G).

#### 3.2.2. En Single Knockdowns Do Not Significantly Affect inv mRNA Levels

**Figure 10:**
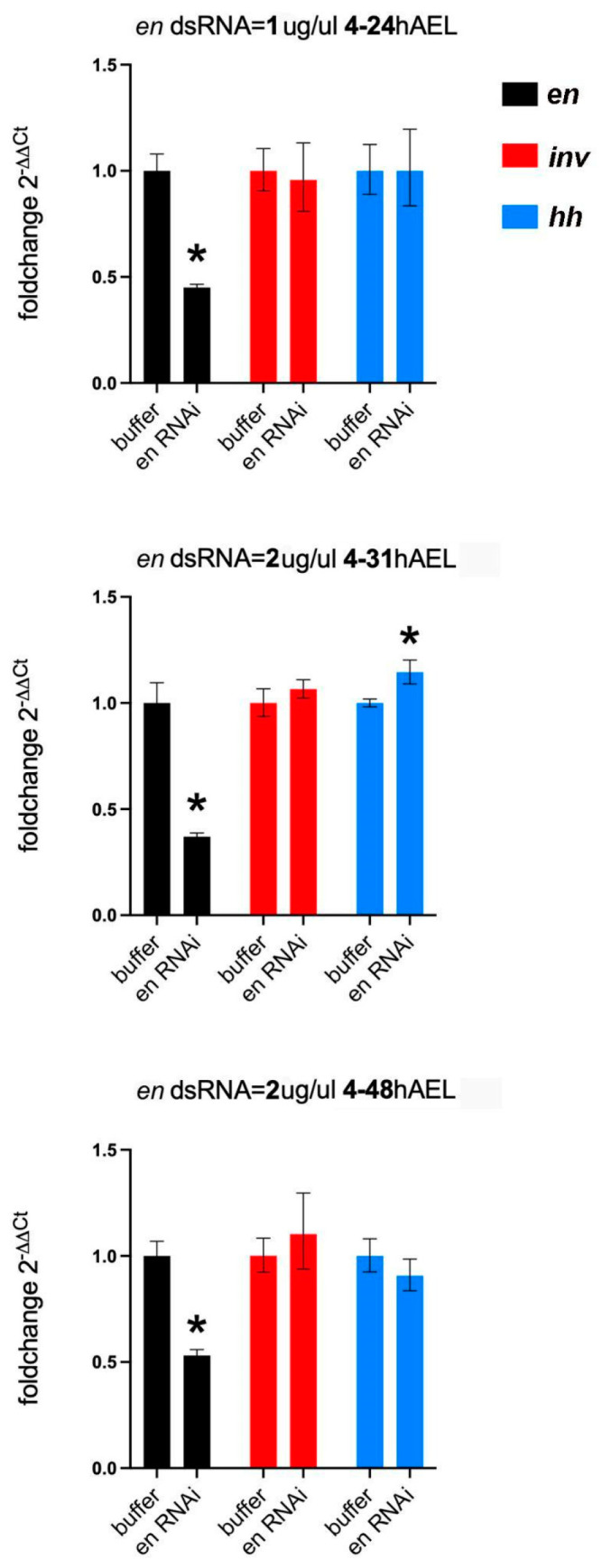
Effects on expression of *inv* and *hh* in *en* knockdowns. *en* knockdown is initiated at 4 h AEL and examined at progressively later stages during which the phenotype is more pronounced. At each time point, *en* expression is reduced (black columns) and *inv* (red) is not affected, but *hh* (blue) levels are statistically increased in the 31 h sample (error bars are SD and significance (*) is *p* < 0.05).

As noted above, *Drosophila en* mutations lead to the loss of *inv* function and some *Drosophila en* enhancer reporters fail to express in *inv* mutants [34,35]. Thus, a potential explanation for the asegmental phenotypes observed at high concentrations of dsRNA injected in the single knockdowns (Figure 9H) could be due to a regulatory interaction between *en* and *inv* in *Tribolium*. To test this idea, we performed qPCR on *en* eRNAi embryos at a series of stages (24, 31, and 48 h AEL) during germband retraction. All stages showed significant knockdown of *en*. We found no evidence of a significant modification of *inv* expression in the *en* knockdowns (Figure 10). We also measured changes in *hh* expression in the *en* knockdowns: *hh* levels would be maintained in the absence of *en* if Inv protein redundantly regulates *hh* in the absence of En protein. *hh* mRNA levels were unchanged at 24 or 48 h AEL but unexpectedly rose slightly at the 31 h AEL timepoint (Figure 10).

## 4. Discussion

### 4.1. Paralog Redundancy—A Model for the Tribolium en/inv Paralogs Acting Synergistically

We find that the *Tribolium en/inv* paralogs have redundant functions in the embryo. Knockdown of either *Tribolium en* or *inv* function results in nearly identical phenotypes. Larvae from both single knockdowns are viable at lower concentrations of dsRNAi-injected but inviable at higher concentrations. Their functional redundancy is further supported by the qPCR analysis of *en* knockdowns. Based on the En-Hh/Wg feedback loop in *Drosophila*, the En protein would be expected to activate *hh* gene expression [42]. Transcription of *hh* did not show a decrease in the *en* knockdowns, consistent with the idea that *inv* can compensate for the loss of *en* function.

We do not find that *en/inv* are fully redundant. A small percentage of the knockdown of either gene alone induces an extreme phenotype with very little discernible pattern in the cuticle (red in Figure 4). A similar phenotype is also seen in the double knockdown, albeit at much higher penetrance than in the single knockdowns. This extreme phenotype does not seem to result from direct or indirect regulation between the paralogs, as is known to occur in *Drosophila* [29,34,35]. Our qPCR analysis of *en* knockdowns did not show a consequent decrease in the transcription of *inv* from 24 to 48 h AEL, as would have been expected from a requirement for either paralog in the continued transcription of the other. An alternate explanation to shared regulation is that the *en/inv* paralogs act synergistically. The double knockdown phenotype is observed when either gene or the combination of both genes, is reduced below a threshold. Similar synergistic effects between paralogs have been reported in *Arabidopsis thaliana* and *Caenorhabditis elegans* [43,44]. In our results, this postulated threshold is reached at lower concentrations of *en* dsRNA injected than *inv* dsRNA. We speculate that the different responses in the *en* and *inv* single knockdowns are primarily due to quantitative differences in expression. Because *inv* mRNA is expressed at much higher levels in the typically developing embryo (Supplemental Figure S3), it is likely more difficult to reach that threshold with *inv*-only dsRNA injections.

One caveat to our model is the absence of the most common double knockdown phenotype (Category D; Figure 3C,D and Figure 7B) from the single knockdowns. This remains unexplained. We note that [32] reports *en* knockdown larvae from pupal RNA that appear to have a similar phenotype, although the bristle pattern was not described.

Redundancy between the *en/inv* paralogs is also seen in *Drosophila*. The *en/inv* redundancy may contribute to both developmental robustness as has been suggested for other gene paralogs [45] as well as the observed evolutionary stability of the segment polarity pathway. While both species have maintained redundant functions of the *en/inv* paralogs, comparing the relative function of each paralog between the two species suggests the degree of genetic redundancy between the two paralogs varies between *Tribolium* and *Drosophila*.

### 4.2. En/Inv Are Redundant for Gnathal and Thoracic Appendage Formation

A requirement for an interaction between *en* and *wg* in the establishment of the proximal-distal axis of the leg has been well documented in flies [46,47,48]. Here we demonstrate that this interaction can be fulfilled redundantly by either *en* or *inv* in *Tribolium*. Gnathal and thoracic appendage formation was absent or severely reduced in the *Tc-en/inv RNAi* larvae but present, albeit at times misshapen, in the single knockdowns (except for the most extremely affected phenotypes). Similarly, appendages were present when *inv* function was knocked down in *Oncopeltus* [14], and in a separate study of *en* knockdowns in *Tribolium* [32]. All these results are consistent with *Tc-en/inv* having redundant roles in larval appendage development, a phenotype that would not be observed in legless *Drosophila* larvae.

The labrum and antennae were present, although sometimes with an irregular shape, in not only single knockdowns but also the double *en/inv* knockdowns that lacked legs. Similar differential effects on labrum and antennae vs. other appendages were reported for Tc-*wg* knockdowns [49] (note that [50] shows a weaker effect) and the Dll expression in the *Tribolium* labrum has been shown to be independent of either Tc-*wg* and Tc-*hh* signaling [50]. Interestingly, antennae are lost in Tc-*hh* pRNAi in both *Tribolium* [51] and *Oncopeltus* [52]. This suggests that Hh signaling is maintained in the antennae by transcriptional activity other than *en/inv.* Antennae have long been considered serially homologous to limbs [53,54,55,56,57] and it has been suggested that the labrum is also appendicular in origin (e.g., [58,59,60]). However, accumulating developmental evidence has been used to argue against considering the head and thoracic appendages as serial homologs [52]. Our evidence provides further developmental genetic support for the proposed lack of serial homology between head and thoracic appendages [52].

### 4.3. En/Hh-Wg—Regulatory Loop

In *Drosophila*, *en* and *inv* expression in the posterior of each segment expression is initiated by the transient activity of the upstream pair-rule genes, then regulated by a positive feedback loop with adjacent *wingless (wg)* expressing cells, mediated by the Hh signal, as described above. While our data do not directly address the feedback loop, careful analysis of the timing of misregulation in *en/inv*, *hh,* or *wg* knockdowns suggests additional players or regulatory interactions are involved in the maintenance of segment boundaries in *Tribolium*. While *wg* expression is lost early in *Tribolium en* knockdowns [32]. En/Inv expression (measured by 4D9 antibody) is maintained throughout gastrulation and germband extension in Tc-*hh* knockdowns [51]. This extended maintenance of the 4D9 stripes in the *hh* knockdowns suggests the presence of additional cues that function to maintain En prior to germband retraction. It is unlikely this is prolonged maintenance from most of the pair-rule genes as their expression has abated by this time. A second ortholog of the pair-rule gene, *sloppy-paired2*, has been shown to have an expression that resembles that of a segment polarity gene in the red flour beetle [61]. It is possible that *slp-2* also functions in the maintenance of segment boundaries during germband extension. We also unexpectedly observed an increase in *hh* expression at 31 h AEL in *en* knockdowns. While this increase in *hh* remains unexplained, it implies a role for En in repressing *hh* expression at the completion of the germband extension. Further experiments are needed to resolve the regulatory interactions between En and *hh* over time and the complete gene regulatory network that maintains segment boundaries in *Tribolium* embryos.

### 4.4. Loss of Intersegmental Cellular Identity vs. Segment Polarity

We have found it curious that while the evidence suggests conservation of at least parts of the Wg-Hh feedback loop that maintains segment polarity, comparative analyses of the knockdown of segment polarity genes *hh* and *wg* in *Tribolium* have emphasized that these knockdowns do not manifest a change in the polarity of the *Tribolium* segmental pattern. *Drosophila en* mutations, while classified as segment polarity mutants, are an exception to the generality of intrasegmental polarity reversals within mutations of the segment polarity genes. The embryonic phenotype of some *Drosophila en* mutants have a significant deletion of the posterior region of even-numbered segments, which led to its initial characterization as a pair-rule gene [24]. However, *Drosophila en* mutants also affects the anterior margin of every segment, thus *en* was unique among the pair-rule genes by having both segmental and pair-rule phenotypes [33]. Stronger *en* mutant alleles result in embryos with apparent segment fusions accompanied by an increase in cell death in portions of the segment [4,62]. While we have not documented cell death in the phenotype, our analysis of the cuticular bristle pattern in the *Tribolium en/inv* double knockdowns demonstrates both segment fusions (Figure 4, Category C/D) and a deletion of a portion of the segment (Figure 4, Category D), consistent with a broadly defined segment polarity phenotype.

### 4.5. Is the Segment Addition Process Disrupted?

One of our original questions based on previously published knockdowns of *Tribolium en* and *Oncopletus inv* was what role, if any, *en/inv* play in the segment addition process in sequentially segmenting insects. Both previous reports document a loss of one-to-many segments in the RNAi knockdowns, which we confirm in our own single and double *en/inv* knockdowns. Our Ubx-abdA expression pattern verifies that, in at least one class of the knockdowns, all trunk segments were made but likely become reduced in size during germband retraction, as is the case in *Drosophila en* mutants [4]. Further analyses of embryo growth over time, with concomitant analyses of cell behaviors and segmentation gene expression, will resolve whether the other classes of embryos preferentially lose segments late in development or fail to add them initially.

We also found that a significant number of double knockdowns fail to form larval cuticle (Figure 8). These embryos have elongated but consist of significantly fewer cells than wild-type or less affected embryos. This failure to grow could result from a primary role for *en/inv* in cell division or a secondary consequence of increased cell death. That the tissue was frequently found coiled in a spiral is consistent with a disruption of cell adhesion, another proposed function of *Drosophila en* [15], and an early disruption of adhesion may have impacted subsequent growth. We speculate that the empty cuticle phenotype observed in both single and double *en/inv* knockdowns (Category E) may also be related to a failure to grow; embryos may have grown sufficiently enough to secrete cuticle but have insufficient growth to complete normal intrasegmental patterning that governs the bristle pattern.

A function for zygotic *en* in organizing the pre-cellular blastoderm, earlier than its function in patterning the posterior compartment, is known in *Drosophila* [62]. *Drosophila en* has also been shown to play a role in the control of growth in the wing disc in a Hh independent mechanism, although in that circumstance its loss of function results in an increase in cell proliferation [63]. Most curious in our phenotypes was the appearance of extensions orthogonal to the primary axis in these embryos. It is possible that the disrupted expression of *en/inv* is causing bifurcations from the primary axis such as that observed as a consequence of the disruption of *en* or *wg* in *Drosophila* imaginal discs [64,65,66]. Thus, as in *Drosophila*, *en* likely has more complex roles than maintaining identity in the cells in the posterior of the segment.

## Data Availability

Data is contained within the article.

## Supplementary Materials

The following supporting information can be downloaded at: https://www.mdpi.com/article/10.3390/insects14080691/s1 Figure S1: *Phylogenetic Tree of en/inv Ensembl hits*. PhylML tree with bootstrap values made with ClustalW based on sequence alignment of *en* or *inv*-like hits from Ensembl aligned with putative orthologs from NCBI Blast hits; Figure S2: *Cycle number of the H3 reference gene at different embryonic stages in Tribolium*. Each timepoint represents the unmodified H3 cycle number from the qPCR averaged across both control and experimental trials. We found in this experiment (and many additional qPCR trials) that the H3 gene gave very consistent cycle numbers across stages and across trials; Figure S3: *Relative expression levels of en and inv mRNA during the first 24 h of development*. *inv* mRNA (red) has a slightly earlier onset of expression, a greater rate of increase, and ultimately reaches roughly 4× the level of expression than en mRNA (blue). At 30 °C at 10 h AEL the embryo is in the later blastoderm, when no stripes of En/Inv protein are detectable, by 13 h AEL the germband has formed and is fully extended, with a full complement of 10 abdominal segments by 24 h. These data are from unpublished work describing the wild-type *Tribolium* transcriptome at the onset of gastrulation through the end of segmentation. Source: [67,68,69].
