# Supplementary material for: Functional Divergence of the Tribolium castaneum engrailed and invected Paralogs
Source: Insects. 2023 Aug 4;14(8):691. doi: 10.3390/insects14080691 (PMC10455198; doi:10.3390/insects14080691)
Supplement: Supplementary file 1 [file insects-14-00691-s001.zip › insects-2477090-supplementary.pdf]

insects MDPI

**Supplementary Data**

**Supplementary Notes on identifying *engrailed* and *invected* orthologs**

### *Identifying and Differentiating* Oncopeltus fasciatus engrailed *and* invected *Homologs*

Angelini and Kaufman (2005 [14]) reported the knockdown of *Oncopeltus en* function using GenBank: AAR23150.1 (personal communication, Angelini). At that time, it was not known that the *Oncopeltus* genome harbored both *en* and *inv* orthologs. In their analysis of the hexapod *engrailed* family, [24] identified AAR23150.1 as *en*. Subsequent to sequencing the *Oncopeltus* genome, AAR23150.1 was reclassified as an *inv* ortholog (OFAS025056_*inv* )[67] and Angelini, personal communication), which they find encodes a diagnostic "RS-motif" from a single, small internal exon, and which they validated by amplification from cDNA, and confirmed by its position in the tail-to-tail oriented *en/inv* tandem duplication. OFAX025151_en was identified as the *engrailed* ortholog, present in the opposite orientation in the tandem duplication [67].

### *Identifying and Differentiating Tribolium castaneum* engrailed *and* invected *Homologs*

Searching for *engrailed* and *invected* in the Ensembl Metazoa *Tribolium castaneum* genome (*T.cas* 5.2) returns several hits for both genes: *engrailed* is associated with three results: TC008952, TC009896, and TC033532. *invected* is associated with two results: TC016368, and TC001164. BLAST analyses revealed that all these genes share significant sequence identity at the amino acid level within their homeodomains (except for TC016368, which shares a cystine-knot cytokine domain with *invected*). Other databases, NCBI protein or nucleotide searches also return variable responses to a query for *Tribolium en* or *inv*. We referred to the previous in-depth phylogenetic analyses of the hexapod *engrailed/invected* gene family [28] that had classified TC008952 as *engrailed* and TC009896 as *invected*. Their relative positions in the conserved tandem duplication in the genome further confirmed their relative orthologies. The remaining genes that return as *en/inv* hits were identified via additional BLAST analyses: TC033532 is identified in NCBI as the *Tribolium* Bar-H1 gene and ortholog of CG13424 *Drosophila lateral muscles scarcer*; TC016368 is identified in NCBI as the *Tribolium spatzle* 6 gene, and an ortholog of CG9196 *Drosophila spatzle 6*; TC001164 is predicted to be *Tribolium ceh-30* and an ortholog of the *C.elegans ceh-19* homeobox gene. BLAST results were confirmed via phylogenetic analysis (Supplementary Figure 1). Sequences used in earlier research focused on TC009896 [68] originally identified as *engrailed* but established in [24] as *invected*. We include this discussion simply to increase awareness of the challenges of automated genome annotation for classifying homeodomain proteins, and in particular *engrailed* gene family members.

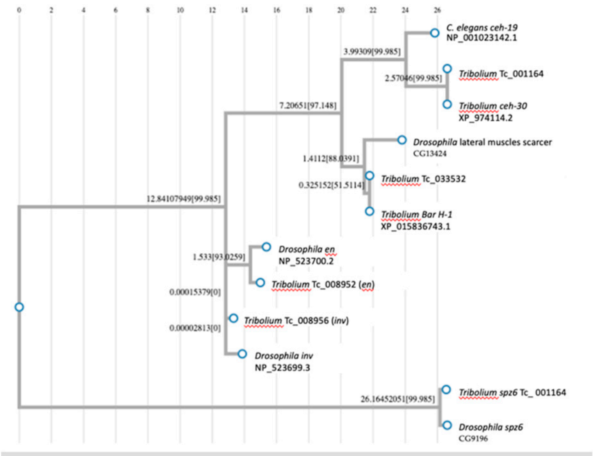


Supplemental Figure S1. *Phylogenetic Tree of en/inv Ensembl hits*
PhylML tree with bootstrap values made with ClustalW based on sequence alignment of *en* or *inv*-like hits from Ensembl aligned with putative orthologs from NCBI Blast hits.

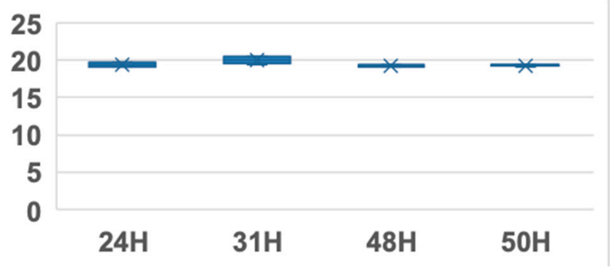


Supplemental Figure S2: *Cycle number of the H3 reference gene at different embryonic stages in Tribolium.* Each timepoint represents the unmodified H3 cycle number from the qPCR averaged across both control and experimental trials. We found in this experiment (and many additional qPCR trials) that the H3 gene gave very consistent cycle numbers across stages and across trials.

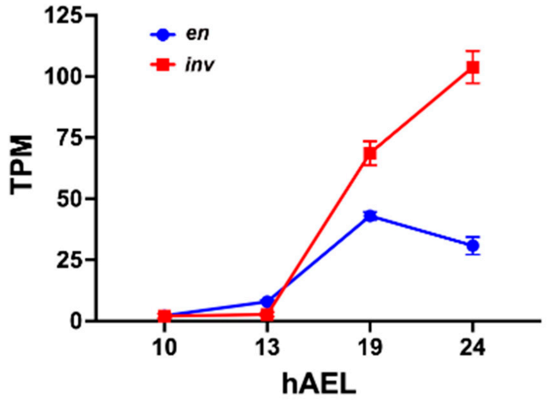


Supplemental Figure S3: *Relative expression levels of en and inv mRNA during the first 24 hours of development. inv* mRNA (red) has a slightly earlier onset of expression, a greater rate of increase, and ultimately reaches roughly 4x the level of expression than *en* mRNA (blue). At 30°C at 10h AEL the embryo is in later blastoderm, when no stripes of En/Inv protein are detectable, by 13hrAEL the germband has formed, and is fully extended, with a full complement of 10 abdominal segments by 24 hrs. These data are from unpublished work describing the wild type *Tribolium* transcriptome at the onset of gastrulation through the end of segmentation [69].
